# Crystal structure of 5′′-benzyl­idene-1′-methyl-4′-phenyl­tri­spiro­[ace­naphthyl­ene-1,2′-pyrrolidine-3′,1′′-cyclo­hexane-3′′,2′′′-[1,3]dioxane]-2,6′′-dione

**DOI:** 10.1107/S2056989016002875

**Published:** 2016-02-20

**Authors:** Kuppan Chandralekha, Deivasigamani Gavaskar, Adukamparai Rajukrishnan Sureshbabu, Srinivasakannan Lakshmi

**Affiliations:** a Research Department of Physics, S. D. N. B. Vaishnav College for Women, Chromepet, Chennai 600 044, India; b Department of Organic Chemistry, University of Madras, Guindy Campus, Chennai 600 025, India

**Keywords:** crystal structure, tris­piropyrrolidines, ace­naphthyl­ene, spiro­cyclo­hexa­nones, dioxalane

## Abstract

In the title compound, C_36_H_31_NO_4_, two spiro links connect the methyl-substituted pyrrolidine ring to the ace­naphthyl­ene and cyclo­hexa­none rings. The cyclo­hexa­none ring is further connected to the dioxalane ring by a third spiro junction. The five-membered ring of the ace­naphthylen-1-one ring system adopts a flattened envelope conformation with the ketonic C atom as flap, whereas the dioxalane and pyrrolidine rings each have a twist conformation. The cyclo­hexa­none ring assumes a boat conformation. Three intra­molecular C—H⋯O hydrogen bonds involving both ketonic O atoms as acceptors are present. In the crystal, C—H⋯O hydrogen bonds connect centrosymmetrically related mol­ecule into chains parallel to the *b* axis, forming rings of *R*_2_^2^(10)and *R*_2_^2^(8) graph-set motifs.

## Abstract 2

In the title tris­piro compound, both the methyl-substituted pyrrolidine and dioxalane rings adopt a twist conformation. The cyclo­penta­none ring of the acenapthylen-1-one system adopts flattened envelope conformation, and the cyclo­hexa­none attached to the dioxalane ring adopts boat conformation. In the crystal, centrosymmetrically related mol­ecules are linked into dimers forming rings of 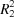
(10) graph-set motif, which are further connected into chains parallel to the *b* axis by C—H⋯O contacts forming rings of 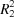
(8) graph-set motif.

## Chemical context   

The biological properties of spiro compounds containing cyclic structures are evident from their presence in many natural products (Molvi *et al.*, 2014 ▸). This class of compounds possesses pharmacological and therapeutic properties which play a fundamental role in biological processes. Several spiro compounds show diverse biological activities such as anti­cancer (Chin *et al.*, 2008 ▸), anti­bacterial (van der Sar *et al.*, 2006 ▸), anti­convulsant (Obniska & Kaminski, 2006 ▸), anti­microbial (Pawar *et al.*, 2009 ▸), anti­tuberculosis (Chande *et al.*, 2005 ▸), anti-oxidant (Sarma *et al.*, 2010 ▸) and pain-relief agents (Frank *et al.*, 2008 ▸). Some spiro compounds are used as pesticides (Wei *et al.*, 2009 ▸) and laser dyes (Kreuder *et al.*, 1999 ▸). They are also used as electroluminescent devices (Lupo *et al.*, 1998 ▸). The spiro­pyrrolidine-3,3′-indole ring system is a recurring structural motif in a number of natural products such as vinblastine and yincristrine which act as cytostatics in cancer chemotherapy (Tan *et al.*, 1992 ▸). Spiro pyrrolidines act as inhibitors of human NK-I receptor activity (Kumar, Perumal, Manju *et al.*, 2009 ▸). They are also exhibit anti­microbial (Sureshbabu *et al.*, 2008 ▸), anti­convulsant and neurotoxic properties (Obniska *et al.*, 2006 ▸) and anti­proliferative activities (Almansour *et al.*, 2014 ▸). Acenaphthalyene derivatives are found to have anti-inflammatory (Smith *et al.*, 1979 ▸), anti­microbial (El-Ayaan & Abdel-Aziz, 2005 ▸), anti­fungal (McDavids & Daniels, 1951 ▸), anti­tumor (El-Ayaan *et al.*, 2007 ▸) and insecticidal activities (Chen *et al.*, 2014 ▸). Dioxalane moieties play a significant role in stabilizing the mutant HIV-1 RT and nucleoside triphosphate. They successfully act as nucleoside reverse transcriptase inhibitors (NRTIs) (Liang *et al.*, 2006 ▸).
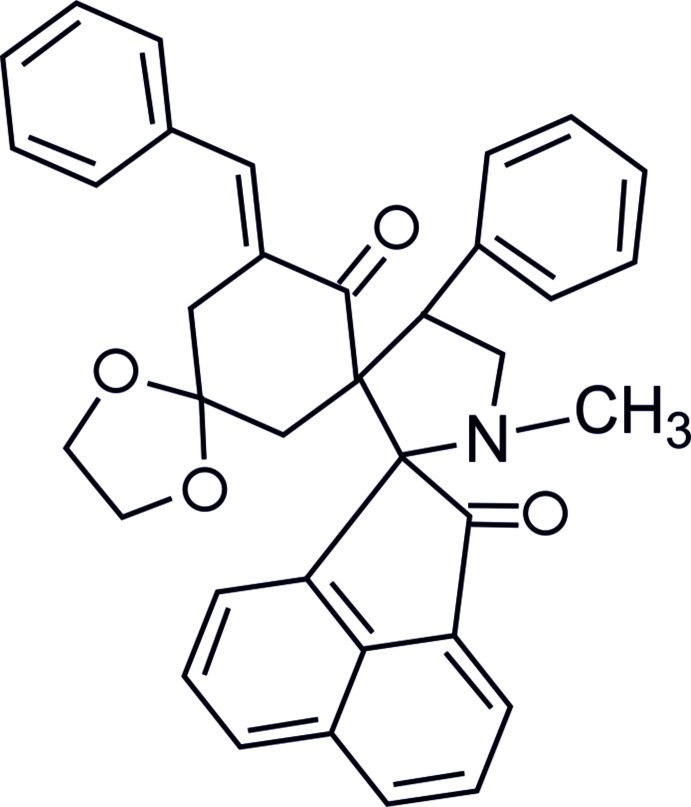


An efficient synthesis of di­spiro­indeno­quinoxaline pyrrolizidine derivatives was accomplished by a one-pot four-component 1,3-dipolar cyclo­addition reaction. A rare di­spiro­heterocyclic compound was synthesized through 1,3-dipolar cyclo­addition of azomethine ylide for the purpose of designing a new class of complex di­spiro­heterocycles with potential biological activities. The reaction yielded a series of spiro [2, 2′] acenaphthen-1′-one-spiro­[3,2′′]indane −1′,3′′-dione-4-aryl pyrrolizidines (Sureshbabu & Raghunathan, 2006 ▸). Novel spiro cyclo­hexa­nones have been synthesized by 1,3-dipolar cyclo­addition of azomethine ylides with anti­tuberculosis activity (Kumar, Perumal, Senthilkumar *et al.*, 2009 ▸). Twelve novel acenaphthene derivatives were reported with anti­tumor activity (Xie *et al.*, 2011 ▸). Geometric *cis*, *trans* isomers derivatives of 2-substituted-1,3-dioxolanes and 2-substituted-1,3-dioxanes have been designed and studied as anti­muscarinic agents (Marucci *et al.*, 2005 ▸). A series of new enanti­omerically pure and racemic 1,3-dioxolanes was synthesized in good yields by the reaction of salicyaldehyde with commercially available diols using a catalytic amount of Mont K10 (Küçük *et al.*, 2011 ▸).

The crystal structures of several biologically significant mono­spiro­pyrrolidines (Chandralekha *et al.*, 2014 ▸) and di­spiro­pyrrolidines (Palani *et al.*, 2006 ▸) have been reported in the literature, but only few reports are available on the crystal structure of tris­piropyrrolidines. In continuation of our work in this field, the crystal structure of title tris­piropyrrolidine is reported on herein.

## Structural commentary   

**Figure 1:**
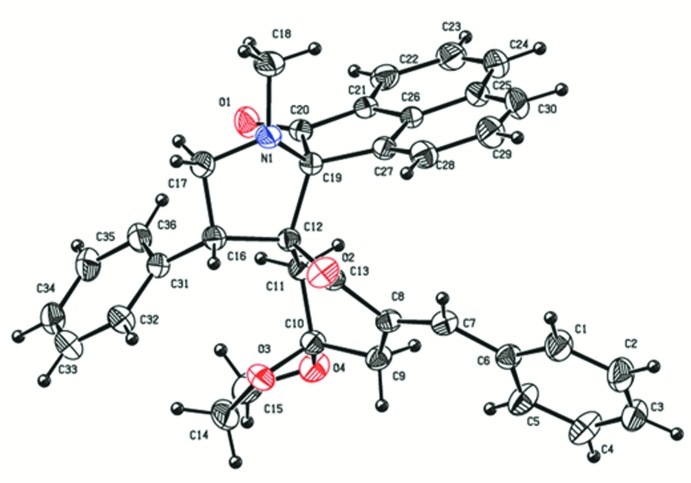
The mol­ecular structure of the title compound, with displacement ellipsoids drawn at the 30% probability level. H atoms are shown as small spheres of arbitrary radius.

In the title compound (Fig. 1 ▸), the methyl-substituted pyrrolidine ring (C12/C16/C17/N1/C19) is in a twist conformation with puckering parameters *q*2 = 0.3809 (18) Å, φ = −66.9 (3)°. The dioxalane ring (C10/O3/C14/C15/O4) also has a twist conformation [*q*2 = 0.327 (2) Å, φ = −58.7 (3)°], while the five-membered ring (C19/C20/C21/C26/C27) of the acenapnthylen-1-one ring system adopts a flattened envelope conformation [*q*2 = 0.0659 (18) Å, φ = −155.6 (16)°]. The six-membered cyclo­hexa­none ring (C8–C13) adopts a boat conformation [*Q*_T_ = 0.616 (2) Å, θ = 75.36 (19)°, φ = 141.65 (18)°]. The least-squares mean plane through the pyrrolidine ring forms dihedral angles of 87.86 (6), 73.34 (7) and 87.81 (6)° with the mean planes of the attached benzene, cyclo­hexa­none and cyclo­penta­none ring, respectively. The mean planes through the cyclo­hexa­none and dioxalane rings form a dihedral angle of 77.99 (8)°. Bond lengths and angles are not unusual and in good agreement with the recently reported values of a related tris­piropyrrolidine compound (Chandralekha *et al.*, 2015 ▸). Three intra­molecular C—H⋯O hydrogen bonds (Table 1 ▸) are present, involving both ketonic O atoms as acceptors.

## Supra­molecular features   

**Figure 2:**
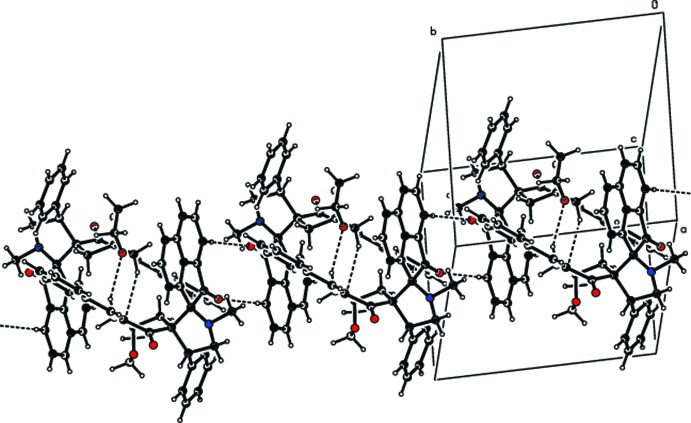
Partial crystal packing of the title compound showing the formation of a mol­ecular chain parallel to the *b* axis through C—H⋯O hydrogen bonds (dashed lines).

In the crystal, centrosymmetrically-related mol­ecules are linked into dimers forming rings of 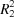
(10) graph-set motif. The dimers are further connected by C—H⋯O contacts forming rings of 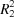
(8) graph-set motif, producing chains parallel to the *b* axis (Fig. 2 ▸).

## Synthesis and crystallization   

An equimolar mixture of 7,9-bis [(*E*)-benzyl­idine)]-1,4-dioxo-spiro­[4,5]decane-8-ones (1 mmol) and sacrosine in methanol (25-30 ml) was refluxed for 4 h. After the completion of the reaction as indicated by TLC, the solid precipitate was filtered and washed with methanol to give the pure tris­piropyrrolidine derivative. Single crystals suitable for the X-ray diffraction analysis were obtained by slow evaporation of the solvent at room temperature.

## Refinement   

Crystal data, data collection and structure refinement details are summarized in Table 2 ▸. All H atoms were placed in calculated positions, with C—H = 0.93–0.98 Å and refined using a riding model approximation, with *U*_iso_(H) = 1.2*U*_eq_(C) or 1.5*U*_eq_(C) for methyl H atoms. A rotating model was applied to the methyl groups.

## Supplementary Material

Crystal structure: contains datablock(s) I. DOI: 10.1107/S2056989016002875/rz5182sup1.cif

Structure factors: contains datablock(s) I. DOI: 10.1107/S2056989016002875/rz5182Isup2.hkl

Supporting information file. DOI: 10.1107/S2056989016002875/rz5182Isup3.cml

CCDC reference: 1454097

Additional supporting information:  crystallographic information; 3D view; checkCIF report

## Figures and Tables

**Table 1:** Hydrogen-bond geometry (Å, °)

*D*—H⋯*A*	*D*—H	H⋯*A*	*D*⋯*A*	*D*—H⋯*A*
C9—H9*A*⋯O4^i^	0.97	2.47	3.352 (3)	152
C17—H17*A*⋯O1	0.97	2.52	3.052 (2)	114
C22—H22⋯O1^ii^	0.93	2.44	3.291 (2)	153
C28—H28⋯O2	0.93	2.59	3.199 (3)	123
C36—H36⋯O1	0.93	2.31	3.174 (3)	155

Symmetry codes: (i) 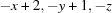; (ii) 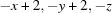.

**Table 2:** Experimental details

Crystal data	
Chemical formula	C_36_H_31_NO_4_
*M*_r_	541.62
Crystal system, space group	Triclinic, *P* 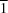
Temperature (K)	293
*a*, *b*, *c* (Å)	10.8861 (4), 11.4899 (4), 11.9171 (4)
α, β, γ (°)	83.83 (1), 65.253 (8), 86.397 (10)
*V* (Å^3^)	1345.60 (12)
*Z*	2
Radiation type	Mo *K*α
μ (mm^−1^)	0.09
Crystal size (mm)	0.30 × 0.25 × 0.20

Data collection	
Diffractometer	Bruker Kappa APEXII CCD
Absorption correction	Multi-scan (*SADABS*; Bruker, 2004 ▸)
*T*_min_, *T*_max_	0.710, 0.746
No. of measured, independent and observed [*I* > 2σ(*I*)] reflections	33777, 4744, 3465
*R*_int_	0.031
(sin θ/λ)_max_ (Å^−1^)	0.595

Refinement	
*R*[*F* ^2^ > 2σ(*F* ^2^)], *wR*(*F* ^2^), *S*	0.038, 0.122, 1.09
No. of reflections	4744
No. of parameters	372
H-atom treatment	H-atom parameters constrained
Δρ_max_, Δρ_min_ (e Å^−3^)	0.16, −0.16

Computer programs: *APEX2* and *SAINT* (Bruker, 2004 ▸), *SHELXS97* (Sheldrick, 2008 ▸), *SHELXL2014* (Sheldrick, 2015 ▸), *ORTEP-3 for Windows* (Farrugia, 2012 ▸), *PLATON* (Spek, 2009 ▸) and *publCIF* (Westrip, 2010 ▸).

## supplementary crystallographic information

### Crystal data

**Table:**

C_36_H_31_NO_4_	*Z* = 2
*M**_r_* = 541.62	*F*(000) = 572
Triclinic, *P*1	*D*_x_ = 1.337 Mg m^−^^3^
*a* = 10.8861 (4) Å	Mo *K*α radiation, λ = 0.71073 Å
*b* = 11.4899 (4) Å	Cell parameters from 43585 reflections
*c* = 11.9171 (4) Å	θ = 5.0–25.7°
α = 83.83 (1)°	µ = 0.09 mm^−^^1^
β = 65.253 (8)°	*T* = 293 K
γ = 86.397 (10)°	Block, colourless
*V* = 1345.60 (12) Å^3^	0.30 × 0.25 × 0.20 mm

### Data collection

**Table:**

Bruker Kappa APEXII CCD diffractometer	3465 reflections with *I* > 2σ(*I*)
Radiation source: graphite	*R*_int_ = 0.031
bruker axs kappa axes2 CCD Diffractometer scans	θ_max_ = 25.0°, θ_min_ = 2.1°
Absorption correction: multi-scan (*SADABS*; Bruker, 2004)	*h* = −12→12
*T*_min_ = 0.710, *T*_max_ = 0.746	*k* = −13→13
33777 measured reflections	*l* = −14→14
4744 independent reflections	

### Refinement

**Table:**

Refinement on *F*^2^	Hydrogen site location: inferred from neighbouring sites
Least-squares matrix: full	H-atom parameters constrained
*R*[*F*^2^ > 2σ(*F*^2^)] = 0.038	*w* = 1/[σ^2^(*F*_o_^2^) + (0.0586*P*)^2^ + 0.2573*P*] where *P* = (*F*_o_^2^ + 2*F*_c_^2^)/3
*wR*(*F*^2^) = 0.122	(Δ/σ)_max_ = 0.001
*S* = 1.09	Δρ_max_ = 0.16 e Å^−^^3^
4744 reflections	Δρ_min_ = −0.16 e Å^−^^3^
372 parameters	Extinction correction: *SHELXL2014* (Sheldrick, 2015), Fc^*^=kFc[1+0.001xFc^2^λ^3^/sin(2θ)]^-1/4^
0 restraints	Extinction coefficient: 0.0109 (19)

### Special details

**Table:**

Geometry. All e.s.d.'s (except the e.s.d. in the dihedral angle between two l.s. planes) are estimated using the full covariance matrix. The cell e.s.d.'s are taken into account individually in the estimation of e.s.d.'s in distances, angles and torsion angles; correlations between e.s.d.'s in cell parameters are only used when they are defined by crystal symmetry. An approximate (isotropic) treatment of cell e.s.d.'s is used for estimating e.s.d.'s involving l.s. planes.

### Fractional atomic coordinates and isotropic or equivalent isotropic displacement parameters (Å^2^)

**Table:**

	*x*	*y*	*z*	*U*_iso_*/*U*_eq_	
O1	0.84726 (14)	0.91261 (12)	0.05682 (13)	0.0608 (4)	
O2	0.50588 (12)	0.57346 (11)	0.30977 (12)	0.0541 (4)	
O3	0.68858 (14)	0.52854 (12)	−0.03539 (12)	0.0570 (4)	
O4	0.91793 (14)	0.51361 (12)	−0.12148 (10)	0.0575 (4)	
N1	0.59384 (15)	0.83289 (13)	0.27966 (13)	0.0454 (4)	
C1	0.7188 (2)	0.23372 (19)	0.45586 (18)	0.0613 (6)	
H1	0.6532	0.2713	0.5207	0.074*	
C2	0.7788 (3)	0.1317 (2)	0.4816 (2)	0.0742 (7)	
H2	0.7530	0.1012	0.5636	0.089*	
C3	0.8757 (2)	0.07441 (19)	0.3887 (2)	0.0670 (6)	
H3	0.9174	0.0064	0.4068	0.080*	
C4	0.9100 (2)	0.11882 (19)	0.2691 (2)	0.0687 (6)	
H4	0.9746	0.0798	0.2048	0.082*	
C5	0.8507 (2)	0.22040 (17)	0.24213 (19)	0.0612 (6)	
H5	0.8756	0.2490	0.1597	0.073*	
C6	0.75437 (18)	0.28139 (15)	0.33522 (16)	0.0433 (4)	
C7	0.68667 (17)	0.39106 (15)	0.31676 (16)	0.0420 (4)	
H7	0.6202	0.4172	0.3892	0.050*	
C8	0.70355 (16)	0.46033 (14)	0.21391 (15)	0.0378 (4)	
C9	0.80155 (18)	0.43569 (15)	0.08521 (14)	0.0431 (4)	
H9A	0.8915	0.4241	0.0831	0.052*	
H9B	0.7767	0.3638	0.0648	0.052*	
C10	0.80439 (18)	0.53226 (15)	−0.00983 (15)	0.0416 (4)	
C11	0.81188 (17)	0.64916 (15)	0.03375 (14)	0.0381 (4)	
H11A	0.8287	0.7093	−0.0343	0.046*	
H11B	0.8877	0.6472	0.0566	0.046*	
C12	0.68310 (16)	0.68198 (14)	0.14449 (14)	0.0353 (4)	
C13	0.62004 (17)	0.57025 (15)	0.22897 (15)	0.0387 (4)	
C14	0.7340 (3)	0.5253 (2)	−0.1650 (2)	0.0846 (8)	
H14A	0.6836	0.5814	−0.1971	0.102*	
H14B	0.7238	0.4478	−0.1849	0.102*	
C15	0.8790 (3)	0.5564 (2)	−0.21767 (19)	0.0808 (8)	
H15A	0.9309	0.5185	−0.2928	0.097*	
H15B	0.8901	0.6404	−0.2356	0.097*	
C16	0.56971 (17)	0.75410 (16)	0.11499 (16)	0.0414 (4)	
H16	0.4871	0.7084	0.1558	0.050*	
C17	0.5450 (2)	0.86368 (17)	0.18436 (18)	0.0515 (5)	
H17A	0.5942	0.9293	0.1288	0.062*	
H17B	0.4494	0.8844	0.2209	0.062*	
C18	0.6007 (2)	0.93114 (19)	0.3440 (2)	0.0649 (6)	
H18A	0.6368	0.9047	0.4035	0.097*	
H18B	0.5115	0.9639	0.3860	0.097*	
H18C	0.6582	0.9897	0.2851	0.097*	
C19	0.71736 (16)	0.76445 (14)	0.22511 (15)	0.0369 (4)	
C20	0.84408 (18)	0.83941 (15)	0.13886 (16)	0.0400 (4)	
C21	0.95258 (17)	0.81145 (14)	0.18008 (16)	0.0398 (4)	
C22	1.08238 (19)	0.84868 (17)	0.13612 (19)	0.0520 (5)	
H22	1.1199	0.8973	0.0630	0.062*	
C23	1.1570 (2)	0.81119 (19)	0.2049 (2)	0.0642 (6)	
H23	1.2463	0.8341	0.1753	0.077*	
C24	1.1034 (2)	0.7423 (2)	0.3136 (2)	0.0655 (6)	
H24	1.1561	0.7206	0.3572	0.079*	
C25	0.9692 (2)	0.70332 (17)	0.36125 (18)	0.0506 (5)	
C26	0.89807 (17)	0.73763 (14)	0.28903 (15)	0.0388 (4)	
C27	0.76373 (17)	0.70795 (15)	0.32229 (15)	0.0388 (4)	
C28	0.6979 (2)	0.64478 (17)	0.43281 (16)	0.0516 (5)	
H28	0.6076	0.6260	0.4591	0.062*	
C29	0.7674 (3)	0.6081 (2)	0.50699 (18)	0.0656 (6)	
H29	0.7221	0.5635	0.5818	0.079*	
C30	0.8985 (3)	0.6354 (2)	0.47332 (19)	0.0658 (6)	
H30	0.9415	0.6091	0.5245	0.079*	
C31	0.58927 (18)	0.78399 (16)	−0.01796 (17)	0.0443 (4)	
C32	0.5014 (2)	0.74079 (19)	−0.0592 (2)	0.0581 (5)	
H32	0.4321	0.6923	−0.0051	0.070*	
C33	0.5148 (3)	0.7683 (2)	−0.1794 (2)	0.0712 (7)	
H33	0.4533	0.7394	−0.2045	0.085*	
C34	0.6167 (3)	0.8373 (2)	−0.2614 (2)	0.0690 (6)	
H34	0.6269	0.8537	−0.3430	0.083*	
C35	0.7037 (2)	0.8819 (2)	−0.2225 (2)	0.0651 (6)	
H35	0.7736	0.9294	−0.2777	0.078*	
C36	0.6887 (2)	0.85717 (18)	−0.10156 (19)	0.0559 (5)	
H36	0.7471	0.8906	−0.0758	0.067*	

### Atomic displacement parameters (Å^2^)

**Table:**

	*U*^11^	*U*^22^	*U*^33^	*U*^12^	*U*^13^	*U*^23^
O1	0.0743 (10)	0.0539 (8)	0.0669 (9)	−0.0217 (7)	−0.0449 (8)	0.0206 (7)
O2	0.0389 (7)	0.0505 (8)	0.0549 (8)	−0.0014 (6)	−0.0022 (6)	−0.0034 (6)
O3	0.0746 (9)	0.0565 (9)	0.0537 (8)	−0.0048 (7)	−0.0393 (7)	−0.0069 (6)
O4	0.0744 (9)	0.0536 (8)	0.0295 (6)	0.0091 (7)	−0.0074 (6)	−0.0072 (6)
N1	0.0481 (9)	0.0442 (9)	0.0493 (9)	0.0106 (7)	−0.0240 (7)	−0.0177 (7)
C1	0.0773 (15)	0.0592 (13)	0.0438 (11)	0.0070 (11)	−0.0234 (10)	−0.0016 (9)
C2	0.1051 (19)	0.0647 (15)	0.0581 (13)	0.0087 (14)	−0.0431 (14)	0.0053 (11)
C3	0.0782 (15)	0.0480 (12)	0.0836 (16)	0.0034 (11)	−0.0452 (14)	0.0048 (12)
C4	0.0709 (15)	0.0444 (12)	0.0719 (15)	0.0082 (11)	−0.0135 (12)	−0.0002 (11)
C5	0.0709 (14)	0.0433 (11)	0.0507 (12)	0.0067 (10)	−0.0094 (10)	0.0027 (9)
C6	0.0467 (10)	0.0383 (10)	0.0425 (10)	−0.0048 (8)	−0.0164 (8)	−0.0008 (8)
C7	0.0421 (10)	0.0406 (10)	0.0367 (9)	−0.0028 (8)	−0.0094 (8)	−0.0042 (8)
C8	0.0379 (9)	0.0347 (9)	0.0371 (9)	−0.0041 (7)	−0.0112 (7)	−0.0040 (7)
C9	0.0505 (11)	0.0371 (10)	0.0359 (9)	0.0032 (8)	−0.0123 (8)	−0.0056 (7)
C10	0.0485 (10)	0.0416 (10)	0.0318 (9)	0.0020 (8)	−0.0136 (8)	−0.0065 (7)
C11	0.0401 (9)	0.0386 (9)	0.0332 (9)	−0.0001 (7)	−0.0132 (7)	−0.0020 (7)
C12	0.0354 (9)	0.0348 (9)	0.0350 (9)	0.0008 (7)	−0.0141 (7)	−0.0045 (7)
C13	0.0368 (10)	0.0405 (10)	0.0377 (9)	−0.0025 (7)	−0.0133 (8)	−0.0067 (7)
C14	0.139 (3)	0.0786 (17)	0.0646 (15)	0.0387 (17)	−0.0697 (17)	−0.0342 (13)
C15	0.141 (3)	0.0565 (14)	0.0338 (11)	0.0209 (15)	−0.0278 (14)	−0.0083 (10)
C16	0.0383 (9)	0.0424 (10)	0.0469 (10)	0.0024 (8)	−0.0211 (8)	−0.0067 (8)
C17	0.0536 (11)	0.0497 (11)	0.0611 (12)	0.0155 (9)	−0.0329 (10)	−0.0166 (9)
C18	0.0775 (15)	0.0575 (13)	0.0730 (14)	0.0181 (11)	−0.0408 (12)	−0.0322 (11)
C19	0.0393 (9)	0.0357 (9)	0.0371 (9)	0.0006 (7)	−0.0170 (7)	−0.0058 (7)
C20	0.0508 (10)	0.0345 (9)	0.0407 (9)	−0.0027 (8)	−0.0244 (8)	−0.0041 (8)
C21	0.0444 (10)	0.0323 (9)	0.0470 (10)	0.0008 (7)	−0.0223 (8)	−0.0080 (7)
C22	0.0477 (11)	0.0421 (11)	0.0675 (13)	−0.0033 (9)	−0.0242 (10)	−0.0072 (9)
C23	0.0516 (12)	0.0566 (13)	0.0990 (18)	0.0005 (10)	−0.0439 (12)	−0.0151 (12)
C24	0.0692 (14)	0.0612 (14)	0.0915 (17)	0.0121 (11)	−0.0579 (13)	−0.0167 (13)
C25	0.0642 (13)	0.0458 (11)	0.0552 (11)	0.0111 (9)	−0.0373 (10)	−0.0144 (9)
C26	0.0483 (10)	0.0338 (9)	0.0404 (9)	0.0077 (8)	−0.0238 (8)	−0.0114 (7)
C27	0.0456 (10)	0.0384 (9)	0.0325 (9)	0.0036 (8)	−0.0161 (8)	−0.0075 (7)
C28	0.0600 (12)	0.0565 (12)	0.0352 (9)	−0.0020 (9)	−0.0164 (9)	−0.0057 (8)
C29	0.0916 (18)	0.0690 (15)	0.0353 (10)	0.0018 (13)	−0.0271 (11)	0.0001 (9)
C30	0.0944 (18)	0.0678 (14)	0.0519 (12)	0.0149 (13)	−0.0486 (13)	−0.0074 (11)
C31	0.0478 (10)	0.0398 (10)	0.0541 (11)	0.0070 (8)	−0.0301 (9)	−0.0081 (8)
C32	0.0603 (12)	0.0606 (13)	0.0683 (13)	−0.0010 (10)	−0.0404 (11)	−0.0096 (10)
C33	0.0901 (17)	0.0723 (15)	0.0794 (16)	0.0019 (13)	−0.0619 (15)	−0.0128 (13)
C34	0.0984 (18)	0.0633 (14)	0.0622 (14)	0.0162 (13)	−0.0523 (14)	−0.0060 (11)
C35	0.0805 (15)	0.0593 (13)	0.0619 (13)	0.0006 (11)	−0.0402 (12)	0.0111 (10)
C36	0.0655 (13)	0.0526 (12)	0.0631 (13)	−0.0049 (10)	−0.0417 (11)	0.0049 (10)

### Geometric parameters (Å, º)

**Table:**

O1—C20	1.210 (2)	C16—C31	1.511 (2)
O2—C13	1.2137 (19)	C16—C17	1.528 (3)
O3—C14	1.415 (3)	C16—H16	0.9800
O3—C10	1.420 (2)	C17—H17A	0.9700
O4—C15	1.412 (3)	C17—H17B	0.9700
O4—C10	1.412 (2)	C18—H18A	0.9600
N1—C17	1.447 (2)	C18—H18B	0.9600
N1—C19	1.447 (2)	C18—H18C	0.9600
N1—C18	1.453 (2)	C19—C27	1.518 (2)
C1—C2	1.375 (3)	C19—C20	1.573 (2)
C1—C6	1.381 (3)	C20—C21	1.464 (2)
C1—H1	0.9300	C21—C22	1.365 (2)
C2—C3	1.364 (3)	C21—C26	1.392 (2)
C2—H2	0.9300	C22—C23	1.398 (3)
C3—C4	1.362 (3)	C22—H22	0.9300
C3—H3	0.9300	C23—C24	1.360 (3)
C4—C5	1.371 (3)	C23—H23	0.9300
C4—H4	0.9300	C24—C25	1.411 (3)
C5—C6	1.386 (3)	C24—H24	0.9300
C5—H5	0.9300	C25—C26	1.394 (2)
C6—C7	1.462 (3)	C25—C30	1.406 (3)
C7—C8	1.338 (2)	C26—C27	1.400 (2)
C7—H7	0.9300	C27—C28	1.358 (2)
C8—C13	1.490 (2)	C28—C29	1.404 (3)
C8—C9	1.502 (2)	C28—H28	0.9300
C9—C10	1.490 (2)	C29—C30	1.359 (3)
C9—H9A	0.9700	C29—H29	0.9300
C9—H9B	0.9700	C30—H30	0.9300
C10—C11	1.510 (2)	C31—C36	1.379 (3)
C11—C12	1.530 (2)	C31—C32	1.381 (3)
C11—H11A	0.9700	C32—C33	1.380 (3)
C11—H11B	0.9700	C32—H32	0.9300
C12—C13	1.545 (2)	C33—C34	1.360 (3)
C12—C19	1.581 (2)	C33—H33	0.9300
C12—C16	1.583 (2)	C34—C35	1.362 (3)
C14—C15	1.486 (4)	C34—H34	0.9300
C14—H14A	0.9700	C35—C36	1.380 (3)
C14—H14B	0.9700	C35—H35	0.9300
C15—H15A	0.9700	C36—H36	0.9300
C15—H15B	0.9700		
			
C14—O3—C10	107.71 (17)	C17—C16—H16	106.6
C15—O4—C10	105.68 (16)	C12—C16—H16	106.6
C17—N1—C19	107.28 (13)	N1—C17—C16	105.12 (14)
C17—N1—C18	114.20 (15)	N1—C17—H17A	110.7
C19—N1—C18	116.15 (15)	C16—C17—H17A	110.7
C2—C1—C6	121.1 (2)	N1—C17—H17B	110.7
C2—C1—H1	119.5	C16—C17—H17B	110.7
C6—C1—H1	119.5	H17A—C17—H17B	108.8
C3—C2—C1	121.0 (2)	N1—C18—H18A	109.5
C3—C2—H2	119.5	N1—C18—H18B	109.5
C1—C2—H2	119.5	H18A—C18—H18B	109.5
C4—C3—C2	118.7 (2)	N1—C18—H18C	109.5
C4—C3—H3	120.6	H18A—C18—H18C	109.5
C2—C3—H3	120.6	H18B—C18—H18C	109.5
C3—C4—C5	120.8 (2)	N1—C19—C27	111.87 (13)
C3—C4—H4	119.6	N1—C19—C20	113.93 (14)
C5—C4—H4	119.6	C27—C19—C20	100.90 (13)
C4—C5—C6	121.37 (19)	N1—C19—C12	103.00 (13)
C4—C5—H5	119.3	C27—C19—C12	118.24 (13)
C6—C5—H5	119.3	C20—C19—C12	109.36 (12)
C1—C6—C5	116.96 (18)	O1—C20—C21	126.20 (16)
C1—C6—C7	117.30 (17)	O1—C20—C19	124.92 (16)
C5—C6—C7	125.74 (16)	C21—C20—C19	108.76 (14)
C8—C7—C6	131.30 (16)	C22—C21—C26	120.60 (16)
C8—C7—H7	114.4	C22—C21—C20	132.49 (17)
C6—C7—H7	114.4	C26—C21—C20	106.78 (15)
C7—C8—C13	117.37 (15)	C21—C22—C23	117.69 (19)
C7—C8—C9	124.61 (16)	C21—C22—H22	121.2
C13—C8—C9	118.02 (14)	C23—C22—H22	121.2
C10—C9—C8	112.48 (15)	C24—C23—C22	122.25 (19)
C10—C9—H9A	109.1	C24—C23—H23	118.9
C8—C9—H9A	109.1	C22—C23—H23	118.9
C10—C9—H9B	109.1	C23—C24—C25	121.07 (19)
C8—C9—H9B	109.1	C23—C24—H24	119.5
H9A—C9—H9B	107.8	C25—C24—H24	119.5
O4—C10—O3	106.59 (13)	C26—C25—C30	116.33 (19)
O4—C10—C9	108.20 (14)	C26—C25—C24	115.90 (18)
O3—C10—C9	110.29 (15)	C30—C25—C24	127.77 (19)
O4—C10—C11	110.75 (14)	C21—C26—C25	122.38 (17)
O3—C10—C11	110.65 (14)	C21—C26—C27	113.75 (15)
C9—C10—C11	110.27 (14)	C25—C26—C27	123.74 (16)
C10—C11—C12	113.28 (14)	C28—C27—C26	118.09 (16)
C10—C11—H11A	108.9	C28—C27—C19	132.38 (17)
C12—C11—H11A	108.9	C26—C27—C19	109.35 (14)
C10—C11—H11B	108.9	C27—C28—C29	119.40 (19)
C12—C11—H11B	108.9	C27—C28—H28	120.3
H11A—C11—H11B	107.7	C29—C28—H28	120.3
C11—C12—C13	109.58 (13)	C30—C29—C28	122.28 (19)
C11—C12—C19	110.40 (13)	C30—C29—H29	118.9
C13—C12—C19	107.26 (12)	C28—C29—H29	118.9
C11—C12—C16	117.13 (13)	C29—C30—C25	120.12 (19)
C13—C12—C16	108.78 (13)	C29—C30—H30	119.9
C19—C12—C16	103.13 (13)	C25—C30—H30	119.9
O2—C13—C8	120.57 (15)	C36—C31—C32	116.90 (18)
O2—C13—C12	120.24 (15)	C36—C31—C16	123.34 (16)
C8—C13—C12	119.12 (14)	C32—C31—C16	119.72 (17)
O3—C14—C15	104.76 (18)	C33—C32—C31	121.2 (2)
O3—C14—H14A	110.8	C33—C32—H32	119.4
C15—C14—H14A	110.8	C31—C32—H32	119.4
O3—C14—H14B	110.8	C34—C33—C32	120.8 (2)
C15—C14—H14B	110.8	C34—C33—H33	119.6
H14A—C14—H14B	108.9	C32—C33—H33	119.6
O4—C15—C14	102.62 (18)	C33—C34—C35	119.1 (2)
O4—C15—H15A	111.2	C33—C34—H34	120.5
C14—C15—H15A	111.2	C35—C34—H34	120.5
O4—C15—H15B	111.2	C34—C35—C36	120.4 (2)
C14—C15—H15B	111.2	C34—C35—H35	119.8
H15A—C15—H15B	109.2	C36—C35—H35	119.8
C31—C16—C17	111.74 (15)	C31—C36—C35	121.60 (19)
C31—C16—C12	120.01 (14)	C31—C36—H36	119.2
C17—C16—C12	104.64 (13)	C35—C36—H36	119.2
C31—C16—H16	106.6		
			
C6—C1—C2—C3	0.2 (4)	C16—C12—C19—N1	25.69 (15)
C1—C2—C3—C4	−1.5 (4)	C11—C12—C19—C27	−84.50 (17)
C2—C3—C4—C5	1.3 (4)	C13—C12—C19—C27	34.84 (19)
C3—C4—C5—C6	0.2 (4)	C16—C12—C19—C27	149.60 (14)
C2—C1—C6—C5	1.3 (3)	C11—C12—C19—C20	30.10 (18)
C2—C1—C6—C7	−179.1 (2)	C13—C12—C19—C20	149.44 (13)
C4—C5—C6—C1	−1.5 (3)	C16—C12—C19—C20	−95.80 (15)
C4—C5—C6—C7	179.0 (2)	N1—C19—C20—O1	−49.5 (2)
C1—C6—C7—C8	176.3 (2)	C27—C19—C20—O1	−169.53 (17)
C5—C6—C7—C8	−4.2 (3)	C12—C19—C20—O1	65.1 (2)
C6—C7—C8—C13	−177.51 (17)	N1—C19—C20—C21	126.69 (15)
C6—C7—C8—C9	1.7 (3)	C27—C19—C20—C21	6.65 (16)
C7—C8—C9—C10	−176.22 (17)	C12—C19—C20—C21	−118.69 (15)
C13—C8—C9—C10	3.0 (2)	O1—C20—C21—C22	−5.9 (3)
C15—O4—C10—O3	28.40 (19)	C19—C20—C21—C22	177.94 (18)
C15—O4—C10—C9	147.01 (17)	O1—C20—C21—C26	169.93 (18)
C15—O4—C10—C11	−92.02 (18)	C19—C20—C21—C26	−6.18 (18)
C14—O3—C10—O4	−8.6 (2)	C26—C21—C22—C23	−0.7 (3)
C14—O3—C10—C9	−125.79 (17)	C20—C21—C22—C23	174.71 (18)
C14—O3—C10—C11	111.93 (17)	C21—C22—C23—C24	−1.6 (3)
C8—C9—C10—O4	167.52 (14)	C22—C23—C24—C25	1.2 (3)
C8—C9—C10—O3	−76.25 (18)	C23—C24—C25—C26	1.4 (3)
C8—C9—C10—C11	46.3 (2)	C23—C24—C25—C30	−177.8 (2)
O4—C10—C11—C12	172.25 (13)	C22—C21—C26—C25	3.4 (3)
O3—C10—C11—C12	54.28 (18)	C20—C21—C26—C25	−173.06 (16)
C9—C10—C11—C12	−68.01 (19)	C22—C21—C26—C27	179.57 (16)
C10—C11—C12—C13	34.08 (18)	C20—C21—C26—C27	3.11 (19)
C10—C11—C12—C19	152.01 (14)	C30—C25—C26—C21	175.62 (17)
C10—C11—C12—C16	−90.41 (18)	C24—C25—C26—C21	−3.6 (3)
C7—C8—C13—O2	−33.8 (2)	C30—C25—C26—C27	−0.2 (3)
C9—C8—C13—O2	146.87 (17)	C24—C25—C26—C27	−179.42 (17)
C7—C8—C13—C12	143.18 (16)	C21—C26—C27—C28	−174.28 (16)
C9—C8—C13—C12	−36.1 (2)	C25—C26—C27—C28	1.8 (3)
C11—C12—C13—O2	−167.12 (15)	C21—C26—C27—C19	1.4 (2)
C19—C12—C13—O2	73.01 (19)	C25—C26—C27—C19	177.50 (15)
C16—C12—C13—O2	−37.9 (2)	N1—C19—C27—C28	48.5 (3)
C11—C12—C13—C8	15.8 (2)	C20—C19—C27—C28	170.00 (19)
C19—C12—C13—C8	−104.02 (16)	C12—C19—C27—C28	−70.9 (2)
C16—C12—C13—C8	145.07 (15)	N1—C19—C27—C26	−126.33 (15)
C10—O3—C14—C15	−13.4 (2)	C20—C19—C27—C26	−4.83 (17)
C10—O4—C15—C14	−35.8 (2)	C12—C19—C27—C26	114.29 (16)
O3—C14—C15—O4	30.2 (2)	C26—C27—C28—C29	−2.3 (3)
C11—C12—C16—C31	2.1 (2)	C19—C27—C28—C29	−176.79 (18)
C13—C12—C16—C31	−122.82 (16)	C27—C28—C29—C30	1.3 (3)
C19—C12—C16—C31	123.53 (16)	C28—C29—C30—C25	0.5 (3)
C11—C12—C16—C17	−124.29 (16)	C26—C25—C30—C29	−1.0 (3)
C13—C12—C16—C17	110.81 (15)	C24—C25—C30—C29	178.2 (2)
C19—C12—C16—C17	−2.85 (17)	C17—C16—C31—C36	57.8 (2)
C19—N1—C17—C16	40.07 (19)	C12—C16—C31—C36	−65.2 (2)
C18—N1—C17—C16	170.29 (16)	C17—C16—C31—C32	−119.55 (19)
C31—C16—C17—N1	−152.50 (15)	C12—C16—C31—C32	117.45 (19)
C12—C16—C17—N1	−21.15 (19)	C36—C31—C32—C33	1.1 (3)
C17—N1—C19—C27	−169.24 (14)	C16—C31—C32—C33	178.67 (19)
C18—N1—C19—C27	61.6 (2)	C31—C32—C33—C34	1.2 (4)
C17—N1—C19—C20	77.11 (17)	C32—C33—C34—C35	−1.9 (4)
C18—N1—C19—C20	−52.0 (2)	C33—C34—C35—C36	0.2 (3)
C17—N1—C19—C12	−41.23 (17)	C32—C31—C36—C35	−2.9 (3)
C18—N1—C19—C12	−170.35 (15)	C16—C31—C36—C35	179.70 (18)
C11—C12—C19—N1	151.59 (13)	C34—C35—C36—C31	2.2 (3)
C13—C12—C19—N1	−89.07 (15)		

### Hydrogen-bond geometry (Å, º)

**Table:**

*D*—H···*A*	*D*—H	H···*A*	*D*···*A*	*D*—H···*A*
C9—H9*A*···O4^i^	0.97	2.47	3.352 (3)	152
C17—H17*A*···O1	0.97	2.52	3.052 (2)	114
C22—H22···O1^ii^	0.93	2.44	3.291 (2)	153
C28—H28···O2	0.93	2.59	3.199 (3)	123
C36—H36···O1	0.93	2.31	3.174 (3)	155

Symmetry codes: (i) −*x*+2, −*y*+1, −*z*; (ii) −*x*+2, −*y*+2, −*z*.
